# eIF2β is critical for eIF5-mediated GDP-dissociation inhibitor activity and translational control

**DOI:** 10.1093/nar/gkw657

**Published:** 2016-07-25

**Authors:** Martin D. Jennings, Christopher J. Kershaw, Christopher White, Danielle Hoyle, Jonathan P. Richardson, Joseph L. Costello, Ian J. Donaldson, Yu Zhou, Graham D. Pavitt

**Affiliations:** Faculty of Biology Medicine and Health, The University of Manchester, Manchester, M13 9PT, UK

## Abstract

In protein synthesis translation factor eIF2 binds initiator tRNA to ribosomes and facilitates start codon selection. eIF2 GDP/GTP status is regulated by eIF5 (GAP and GDI functions) and eIF2B (GEF and GDF activities), while eIF2α phosphorylation in response to diverse signals is a major point of translational control. Here we characterize a growth suppressor mutation in eIF2β that prevents eIF5 GDI and alters cellular responses to reduced eIF2B activity, including control of *GCN4* translation. By monitoring the binding of fluorescent nucleotides and initiator tRNA to purified eIF2 we show that the eIF2β mutation does not affect intrinsic eIF2 affinities for these ligands, neither does it interfere with eIF2 binding to 43S pre-initiation complex components. Instead we show that the eIF2β mutation prevents eIF5 GDI stabilizing nucleotide binding to eIF2, thereby altering the off-rate of GDP from eIF2•GDP/eIF5 complexes. This enables cells to grow with reduced eIF2B GEF activity but impairs activation of *GCN4* targets in response to amino acid starvation. These findings provide support for the importance of eIF5 GDI activity *in vivo* and demonstrate that eIF2β acts in concert with eIF5 to prevent premature release of GDP from eIF2γ and thereby ensure tight control of protein synthesis initiation.

## INTRODUCTION

In eukaryotic protein synthesis initiation the guanosine-5′-triphosphate (GTP) binding protein eIF2 delivers initiator tRNA (Met–tRNA_i_) to the small ribosomal subunit as part of the 43S pre-initiation complex (PIC). Following mRNA selection eIF2 also assists with AUG codon selection during scanning to ensure accurate protein synthesis (1,2). The affinity of eIF2 for Met–tRNA_i_ is dictated by its nucleotide status as eIF2•GTP has high affinity for Met–tRNA_i_, while eIF2•GDP does not (3). Upon GTP hydrolysis and phosphate release eIF2•GDP loses affinity for tRNA and leaves the PIC (4). Hence eIF2 must reacquire GTP to facilitate continued rounds of translation initiation. The release of GDP from eIF2 is promoted by the guanine nucleotide exchange factor (GEF) eIF2B; an important mediator of protein synthesis control (5).

eIF2B GEF activity is specifically inhibited to modulate protein synthesis initiation rates in response to diverse cues across all eukaryotes studied. Signals include nutritional imbalances (6), endoplasmic reticulum stress and modulation of learning and memory (7–11), which activate one of four protein kinases that each phosphorylate eIF2α at serine 51 eIF2(αP). This converts eIF2 into a competitive inhibitor of eIF2B GEF (12–14), by promoting tight binding of eIF2α to the αβδ subunits of eIF2B (15), presumably in a manner such that the GEF domain at the eIF2Bϵ C-terminus (16) cannot access eIF2γ-bound GDP for nucleotide exchange. Elevated levels of eIF2(αP) lowers overall translation and activates translation of a subset of translationally controlled genes such as those bearing upstream open reading frames (uORFs) including *GCN4* in yeast and ATF4 in mammals (17,18). Glia appear particularly sensitive to reductions in eIF2B activity because missense mutations in eIF2B subunits cause the fatal neurological disorder Leukoencephalopathy with vanishing white matter (VWM; OMIM #603896) (19).

Hydrolysis of eIF2•GTP within the PIC is promoted by the GTPase activating protein (GAP) eIF5 (4), and upon AUG-codon recognition, eIF5/eIF2•GDP complexes dissociate prior to 60S joining (20). eIF5/eIF2•GDP complexes represent an abundant complex in yeast cells (21,22). eIF5 has a second function within this complex to prevent spontaneous release of GDP from eIF2, termed GDP dissociation inhibitor (GDI) activity (23). eIF5 GDI antagonizes eIF2B GEF and is necessary for tight control of translation by eIF2(αP) by ensuring that there is minimal eIF2B independent GDP exchange. This GDI function requires the eIF5 C-terminal domain (CTD) and the adjacent upstream linker region (LR). Mutations in either domain that weaken eIF5–eIF2 interactions eliminate GDI activity (23). When eIF2-free active eIF2B is present, it can efficiently displace eIF5 from eIF2•GDP. Consistent with other G protein systems, this additional role for eIF2B is termed a GDI-displacement factor (GDF) function (14). Thus eIF2B GDF activity displaces eIF5 GDI from eIF2•GDP to enable eIF2B GEF and continued rounds of protein synthesis (2,24).

Here we present a molecular analysis of a growth suppressor mutation that spontaneously arose during our studies of eIF2B GEF mutants. We show that it is a novel missense allele within the β subunit of eIF2 (E189K). Biochemical analyses reveal that this mutation does not affect the intrinsic nucleotide, Met–tRNA_i_ or 43S PIC-binding affinities of eIF2. Instead we find that it prevents eIF5 GDI activity. Our genetic studies show that this alters the sensitivity of cells to eIF2B activity and eIF2(αP), thereby affecting the derepression of *GCN4* translation. These results uncover an important role for eIF2β–eIF5 interactions for controlling eIF5 GDI activity, suggesting that eIF2β E189 makes important contributions to this function. They also demonstrate that eIF2β is important for determining the cellular responses to the eukaryote-wide eIF2(αP) translational control mechanism.

## MATERIALS AND METHODS

### Yeast genetics and cell culture

Strains used and generated in this work are derivatives of S288c and are described in Supplementary Table S1. Cell growth was performed at 30°C in standard yeast rich (YPD), synthetic defined minimal (SD) or synthetic complete (SC) media lacking nutrients for plasmid selection. All media have 2% glucose as a carbon source (25). 3-Amino-1,2,4-triazole (3AT) (Fluka) was added where indicated. For serial dilution growth assays, cells were grown to A_600_ = 0.6, diluted to 0.1 and 2–3 μl of 10-fold dilutions were spotted on the agar surface. Plasmid shuffling to select for loss of *URA3* plasmids employed 5-fluoro-orotic acid (5-FOA; Sigma-Aldrich), while loss of *LEU2* plasmids relied on passive loss during unselected growth and subsequent screening. Mating type switching employed *HO* plasmid pAV1621 (pHO1.5) followed by 5-FOA, sporulation, tetrad dissection and mating type testing (25). *gcd6Δ sui3*Δ double deletion strains (GP7216-19) were made by polymerase chain reaction (PCR) amplifying *gcd6Δ::KanMX* cassette from a BY4743 *gcd6Δ::KanMX/GCD6* diploid strain (GP4146) and transforming the DNA fragment into *sui3Δ gcn2Δ* [*SUI3*-Flag *TRP1*] [*GCD6 URA3*] strains to generate G418 resistant cells unable to lose the covering *GCD6* plasmid upon 5-FOA selection.

### Plasmid constructions

Plasmids used are described in Supplementary Table S2. The V5 epitope was added to the N-terminus of eIF2 (*SUI3*) to create pAV2322 in two steps. First tagged *SUI3* was made by custom synthesis (Epoch life Sciences) of a *SUI3* gene 5′ fragment inserting the V5 epitope (GKPIPNPLLGLDST) between the second and third codon. This also introduced a SpeI restriction site. Next XmaI and MluI were used to cut and insert this fragment into the same sites of the high copy plasmid pAV1726 (16) to generate a *LEU2* plasmid with all three eIF2 genes V5-*SUI3, SUI2* and his_6_-*GCD11* each expressed under their native promoters.

The E189K mutation was introduced into pAV2322 and also pAV2443 (KAB258) (26) to create pAV2451 and pAV2452 by Quikchange site-directed mutagenesis (Agilent Technologies) using the oligonucleotides SUI3E189KFOR (GCATAGATCTCCGAAACATTTGATTCAATATCTCTTCGC) and SUI3E189KREV (GAATCAAATGTTTCGGAGATCTATGCAATTTTTCGGCG). Colonies were screened with BspE1 digestion and the *SUI3* ORF in the constructs was verified by Sanger DNA sequence analysis.

### Genomic DNA sequencing

GP3755 and GP3763 genomic DNA was sequenced using a SOLiD4 sequencer (Life Technologies) at the University of Manchester genomics technologies facility using standard kits and protocols. To generate paired-end reads (F3 [start of DNA fragment] = 50 bp and F5 [end of DNA fragment] = 35 bp) the CSFASTA and QUAL files for each sample were converted to FASTQ format using the solid2fastq script (BFAST software) (27,28). The reads were quality filtered using SOLiD_preprocess_filter_v2.pl (29) (settings used -x y -p 3 -q 22 -y y -e 10 -d 9). The reads were mapped to the *Saccharomyces cerevisiae* reference genome ‘sacCer3’ obtained from UCSC (30) (http://genome.ucsc.edu/), which includes the assembled chromosomes, the mitochondrial genome and the two micron plasmid. The F3 reads were mapped using BFAST ‘match’ and the F5 reads were mapped using BFAST + BWA 0.7.0.a ‘bwaaln’. Mapping was completed as single-ended using the F3 reads only, or the individually mapped F3 and F5 reads were both used for paired-end mapping. In both cases the ‘-a 2’ flag of the post-process step was used to obtain uniquely mapping alignments. The F3 reads of properly paired reads were obtained using ‘samtools view -f 66’ (31). SRMA v0.1.15 was used to realign reads to correct for erroneous mapping caused by the presence of INDELs at the end of reads. Variant detection was performed using samtools v0.1.18, using mpileup and bcftools without BAQ computation (32,33). Candidate variants where filtered using vcfutils.pl requiring at least 20 high-quality read matches and a maximum coverage calculated as three or five times the median coverage (including 0 counts). Variants were also filtered to remove any with QUAL (variant quality) <40.

### Verification *sif285-1* is *sui3-E189K*

Genomic DNA was isolated from yeast strains GP3771-4 using standard procedures (25) and the *SUI3* ORF amplified by PCR using primers eIF2betaF1 (ATGTCCTCCGATTTAGCTGC) and eIF2betaR286 (TCACATTCTCCTTCTCTTACC). PCR products were screened by BspE1 digestion and verified by Sanger DNA sequence analysis.

### Sequence alignment

Alignment generated using Clustal X (34) and the following eIF2β sequences (termed EIF2S2, *SUI3* or *TIF212*) from Genbank: *S. cerevisiae* (CAA97959.1), *Candida albicans* (XP_715210.1), *Candida glabrata* (XP_446841.1), *Cryptococcus neoformans* (AAW43820.1), *Schizosaccharomyces pombe* (CAB11076.2), *Caenorhabditis elegans* (CAB00049.1), *Homo sapiens* (CAG33015.1), *Mus musculus* (AAI13767.1), *Arabidopsis thaliana* (AAK29672.1), *Oryza sativa* (NP_001060649.1), *Glycine max* (XP_006575455.1).

### Protein synthesis

The rate of protein synthesis was measured in exponential phase cells in SC-uracil-methionine at 30°C in a water bath. Once cells (10 ml cultures in 50 ml flasks) reached A_600_ = 0.1, 160 μl methionine mix was added (1 μl [^35^S] methionine (1175 Ci/mmol Tran35S-label, MP Biomedicals) in 50 μM methionine). 100 μl samples were removed immediately and every 20 min over a 2 h period and were immediately precipitated with ice-cold 20% trichloroacetic acid (TCA). Total protein incorporation was measured by scintillation counting of TCA-precipitates absorbed onto GF/C filters (Whatman) washed with ice-cold acetone.

For Polysome profile analysis yeast strains were grown to an A_600_ = 0.7 in 100 ml YPD medium. For glucose starvation, cells were collected by rapid centrifugation at room temperature and resuspended in pre-warmed YP for 10 min prior to final harvest. Extracts were prepared in 50 μg/ml cycloheximide and layered onto 15–50% sucrose gradients as described (35). The gradients were sedimented via centrifugation at 40 000 rpm for 2.5 h using a SW41 rotor (Beckman). Sucrose density gradient fractionation was performed on an ISCO gradient fractionator and the A_254_ was measured continuously to give the traces shown.

### β-galactosidase assay

Measurement of β-galactosidase levels from a *HIS4-LacZ* fusion genomically-integrated at *ura3-52* was performed exactly as described (36) using whole protein extracts from cells grown in SC medium lacking histidine and uracil or following addition of 10 mM 3AT for 6 h. Units are nmole *o*-nitrophenyl-β-D-galacto-pyranoside cleaved/min/μg total protein ± SE, *n* = 3.

### FLAG immune precipitation and western blotting

FLAG (Sigma-Aldrich) immune precipitation and western blotting were performed as described recently (23,37,38) using whole cell extracts to capture FLAG-eIF2β and associated proteins. Bound proteins were probed using specific antibodies and quantitative IR western blot detection was performed using IRDye^®^ 800CW goat anti-rabbit IgG or IRDye^®^ 680RD goat anti-mouse IgG with an Odyssey Fc imager (Li-Cor).

### GST pull-down assay

Purified GST-eIF5 or GST alone (200 pmol) and eIF2 (200 pmol) were incubated in 500 μl of interaction buffer (30 mM HEPES pH 7.5, 100 mM KCl, 0.1 mM ethylenediaminetetraacetic acid (EDTA), 1 mM 1,4-dithiothreitol (DTT), 2.5 mM MgCl_2_) with 25 μl of glutathione-Sepharose beads for 1 h at 4°C. Beads were washed three times with 500 μl of interaction buffer and bound proteins were eluted by boiling in protein-sample buffer. Western blotting was performed as above.

### Protein purification

eIF2 was purified from yeast strains GP7145 and GP7146 expressing V5-*sui3-E189K* or the V5-*SUI3* derivatives of eIF2β by sequential nickel affinity, HiTrap Heparin and HiTrap Q sepharose chromatography as described (14). To obtain apo–eIF2 free from nucleotide, eIF2 was dialysed with EDTA (30 mM HEPES, 100 mM KCl, 1 mM DTT, 1 mM EDTA, 10% glycerol, pH 7.4) then with magnesium (30 mM HEPES, 100 mM KCl, 1 mM DTT, 0.1 mM MgCl_2_, 10% glycerol, pH 7.4). GST-eIF5 was purified from *Escherichia coli* as described (23). eIF2B was purified from yeast strain GP5949, as described (39).

### Steady state fluorescence

To assay nucleotide affinity, fluorescence intensity of 100 nM of BODIPY-FL-GDP or BODIPY-FL-GTP (Thermo Fisher Scientific) in 180 μl of assay buffer (30 mM HEPES, 100 mM KCl, 10 mM MgCl_2_, pH 7.4) was measured using a Fluoromax-4 spectrophotometer (Horiba) (490 nm excitation, 509 nm emission). Yeast Met–tRNA_i_ was assayed in a similar manner using 20 nM BOP-N-Met–tRNA_i_ (tRNA probes) but with the addition of 1 mM GTP. Change in fluorescence intensity was measured upon addition of increasing amounts of apo–eIF2, incubating for 5 min at room temperature each time. Each measurement was blanked against a control without nucleotide to account for any affect of eIF2 and data were corrected for dilution effects caused by volume addition and normalized to starting values before being fitted to a single site binding model: y = 1 + [(ΔF_max_ - 1)*(x/(x + *K*_d_))].

### GDP dissociation assay

Fluorescent eIF2•BODIPY-GDP binary complex was formed by incubating apo–eIF2 with a 2× excess of BODIPY-FL-GDP (Thermo Fisher Scientific) for 20 min at room temperature. Excess nucleotide was removed by passing through a G-50 sephadex column (GE Healthcare). Labelling efficiency was calculated to exceed 90%. To measure GDP release, 20 nM eIF2•BODIPY-GDP was quickly mixed with 1 mM of unlabelled GDP (±eIF2B and ±GST-eIF5) in 180 μl of assay buffer (30 mM HEPES, 100 mM KCl, 10 mM MgCl_2_, pH 7.4) and fluorescence intensity was continuously measured using a Fluoromax-4 spectrophotometer (Horiba) (490 nm excitation, 509 nm emission, 0.1 s integration time). Experimental data were fitted to exponential dissociation curves to determine the rate constants (*K*_off_) at each eIF2B concentration. *K*_1/2_ and *K*_max_ values were determined from curve fitting y = [(*K*_max_ × x)/(*K*_1/2_ + x)] + c. Yeast eIF2B was recently shown to be a functional dimer (40), so likely interacts with eIF2 as a 2:2 complex. For consistency with prior studies, eIF2B and eIF2 concentrations stated and equations used refer to 5-subunit and 3-subunit monomeric complexes respectively.

## RESULTS

### *sif285-1* is a spontaneous growth suppressor of eIF2B mutations

**Figure 1.**
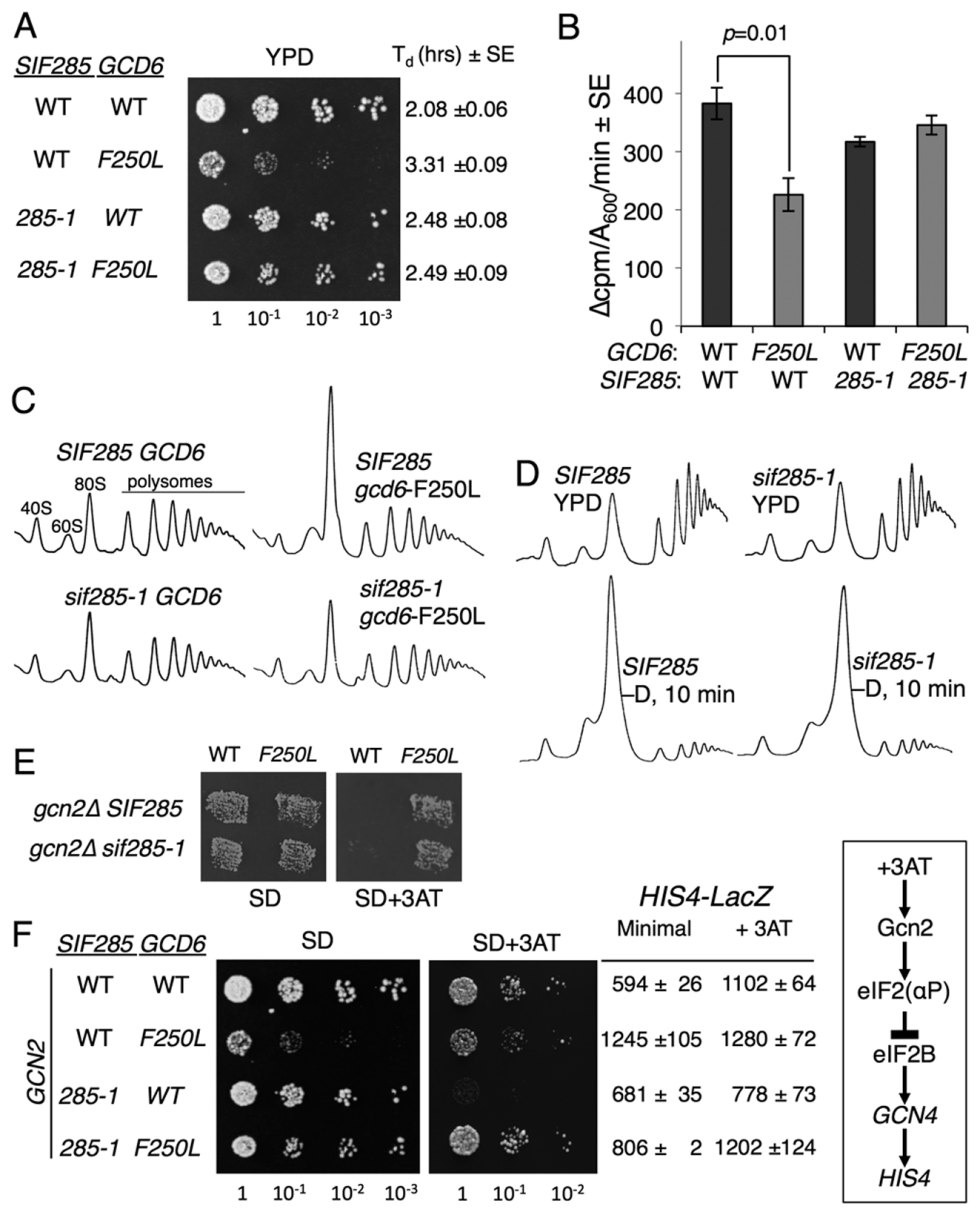
An extragenic spontaneous slow-growth suppressor of eIF2Bϵ mutations. (**A**) *sif285-1* suppresses the slow-growth of the *gcd6-*F250L mutation. Growth of strains GP3771-3774 on YPD. Relevant genotypes and doubling times from liquid cultures are shown. (**B**) Total protein synthesis rates (^35^S-met incorporation) ± SE (*n* = 3). Student's *t*-test indicates only *F250L* differs significantly from the other three strains (*P* < 0.05). (**C** and **D**) Polysome profile analysis for strains shown in (A) grown in YPD or following 10 min incubation in YP lacking glucose (D). Traces show A_254_ recorded by continuous fractionation of 15–50% sucrose gradients. (**E**) Replica printing of the strains shown in panel A on minimal SD ±3AT. (**F**) Growth on SD ±3AT media and β-galactosidase assay of *HIS4-lacZ* for strains shown in (A), but following transformation with a *GCN2* plasmid (pAV1198). Right box summarizes the signalling pathway leading to derepression of *HIS4* expression by amino acid starvation (+3AT).

A spontaneous suppressor of the slow-growth of one yeast eIF2Bϵ mutated strain (*gcd6-F250L*) was noted following routine sub-culture on solid medium (Supplementary Figure S1A). The suppressor-bearing strain carries a deletion of the essential *GCD6* gene and is complemented with a plasmid borne *gcd6-F250L* allele. Following transformation and plasmid shuffling the growth-suppressor strain showed a clear difference in growth rate compared to wild-type (WT) cells when both were combined with the *gcd6-F250L* mutation which grow slowly in the WT background and grow well in the growth-suppressor strain. The unknown mutation was hence initially named *sif285-1* for *s*uppressor of e*IF2B5* and the WT version termed *SIF285* (Figure 1A and Supplementary Figure S1B). This name is used in the description of our analyses here prior to the identification the molecular defect causing the mutation.

Measurements of growth-rates in liquid cultures, total protein synthesis by ^35^S-methionine incorporation and polysome profile analyses showed the defect in eIF2B activity conferred by the F250L mutation affected general protein synthesis in an otherwise WT strain, as expected from previous studies (41). In contrast, these defects were largely suppressed by the presence of the *sif285-1* mutation (Figure 1A–C)*. sif285-1* was also able to restore faster growth to all slow-growing *gcd6* mutations tested (Supplementary Figure S1C), including suppressing the lethality of *gcd6-N249K*, a Gcd6 mutation which retains residual GEF activity that is insufficient to permit growth of otherwise WT cells (41). However, *sif285-1* could not rescue a complete deletion of *GCD6*. Mating to create **a**/α diploid cells showed that the *sif285-1* phenotype was semi-dominant (Supplementary Figure S1D). Sporulation and tetrad dissection showed the fast-growing phenotype segregated 2:2 (Supplementary Figure S1E), indicating that *sif285-1* was likely a single gene alteration.

Mutations in multiple factors can cause a loss of translational control, including mRNA-decay pathway mutants (42). To assess if *sif285-1* is generally resistant to translational control responses, we monitored the initial cell responses to glucose starvation by polysome profile analysis following 10 min glucose withdrawal, as this response is independent of eIF2B inhibition (43). *sif285-1* mutant cell translation remains glucose-withdrawal sensitive, suggesting *sif285-1* suppression has at least some eIF2B specificity (Figure 1D). Because yeast eIF2B mutations constitutively derepress *GCN4* translation and the Gcn4-mediated general amino acid control response (GAAC) independent of Gcn2 (termed a Gcd^–^ phenotype), we assessed resistance of *sif285-1* cells to 3AT, a competitive inhibitor of histidine biosynthesis that activates GAAC via the signalling pathway (box in Figure 1F) (17). *sif285-1* could not prevent the *GCN2* independent 3AT resistance of *gcn2*Δ *gcd6-F250L* cells in a replica plating assay (Figure 1E). To examine if *sif285-1* cells alter the Gcn2-dependent GAAC response, we transformed these cells with a WT *GCN2* plasmid and repeated our growth analyses. *sif285-1 GCN2* cell growth was impaired by 3AT, indicating that *sif285-1* was a GAAC non-derepressible mutant (a Gcn^–^ phenotype, Figure 1F). This observation was confirmed by analysing the levels of β-galactosidase expressed from a genomically integrated *HIS4-LacZ* fusion present in these strains. *HIS4* transcription is derepressed ∼2-fold by GAAC via increased *GCN4* translation in the presence of 3AT (17). As expected, *gcd6-F250L* cells exhibit high unregulated *HIS4* expression. In contrast, *HIS4-LacZ* expression is not derepressed by 3AT treatment in *sif285-1* cells bearing *GCD6*, while regulated *HIS4* expression is restored in *gcd6-F250L sif285-1* double mutant cells (Figure 1F). In summary our data suggest that the *sif285-1* mutation permits cells to grow with reduced requirement for eIF2B that alters the threshold for induction of the GAAC response, but does not confer complete insensitivity to eIF2B. Because mutations in human eIF2B that lead to VWM usually impair eIF2B GEF activity and can cause heightened ATF4 expression (44,45), we decided it would be of interest to characterize the *sif285-1* mutation further. Standard complementation experiments showed the *sif285-1* mutation was not in any of the five eIF2B subunits.

### *sif285-1* is E189K in eIF2β (*SUI3*)

**Figure 2.**
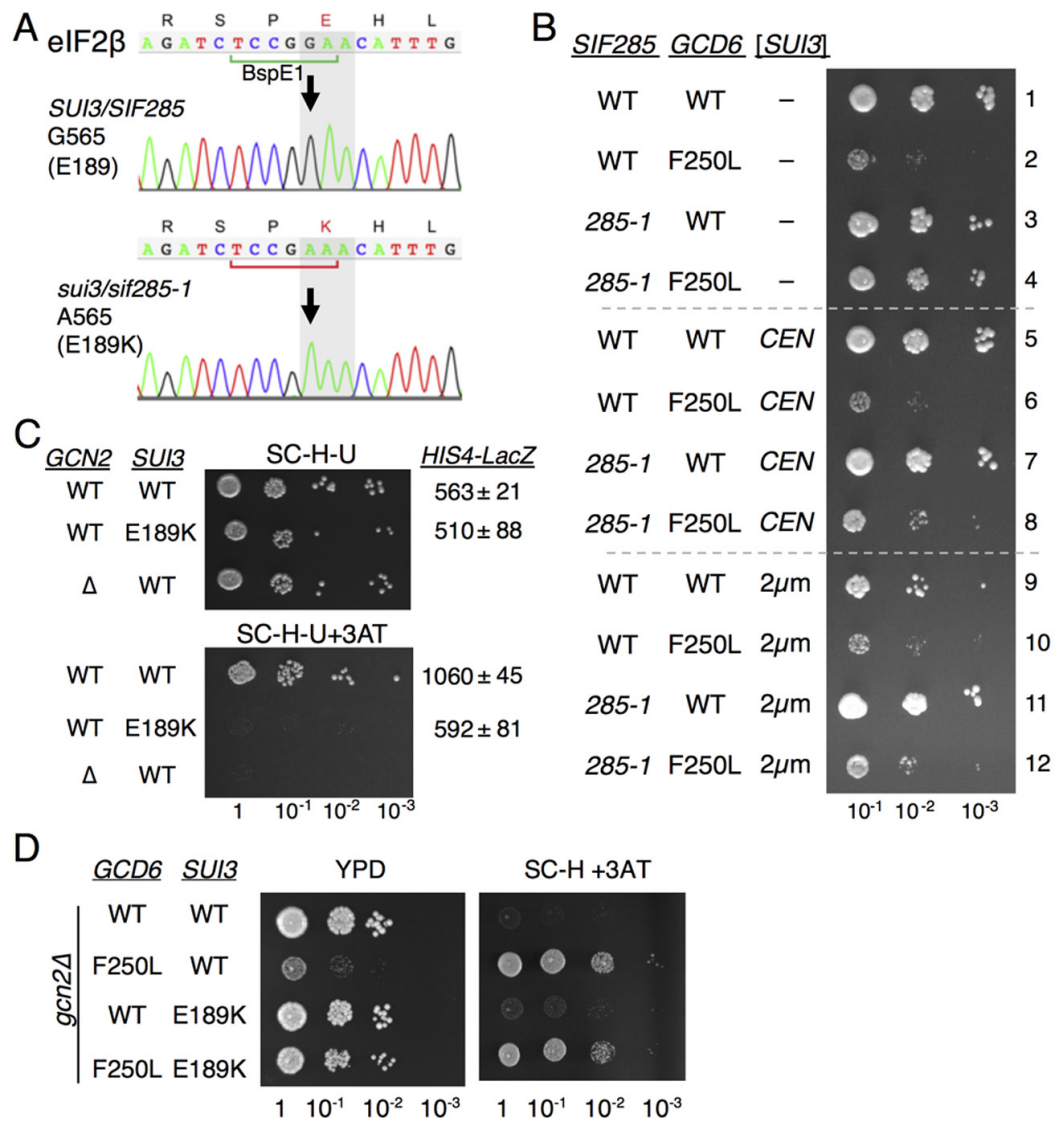
*sif285-1* is the *sui3-E189K* mutation in eIF2β. (**A**) Sanger sequencing confirms *sif285-1* cells (GP3773) contain *sui3*-G565A mutation. (**B**) Growth of strains GP3771-4 transformed with plasmid vector (rows 1–4), *SUI3* low copy (rows 5–8) or *SUI3* high copy (rows 9–12) on SD dropout medium. (**C**) Growth of WT (GP7124: Flag-*SUI3*) and E189K (GP7125 Flag-*SUI3-E189K*) strains transformed with *GCN2* (pAV1198) or a vector control (pAV195) on SC-histidine ±3AT media and *HIS4-LacZ* assays. (**D**) Growth of *gcd6Δ sui3Δ* double deletion strains (GP7216-7219) bearing the indicated alleles of each gene on YPD and SC-histidine +3AT media.

To identify the *sif285-1* defect we isolated genomic DNA from the original *sif285-1* strain (GP3563) and its parent (strain GP3755) and sequenced it by SOLiD4 next generation sequencing (ABI). Following mapping to the reference *S. cerevisiae* full genome (UCSC version *sacCer3*), the reads were screened for single nucleotide variants (SNV) and deletions using standard informatics approaches. SNV analysis revealed only seven clear differences between the WT and *sif285-1* genomes that had at least 20 high-quality mapped reads (Supplementary Table S3). Only two SNVs corresponded to missense mutations; the first change *gcd6-F250L* was known and expected, while the second caused an *E189K* change in *SUI3. SUI3* encodes for the eIF2β subunit: a known interacting partner of Gcd6/eIF2Bϵ (26), and was therefore the likely *sif285-1* candidate. The missense mutation was predicted to eliminate a BspE1 restriction site in *SUI3*. The *SUI3* ORF was amplified by PCR from the strains and both BspE1 digestion (Supplementary Figure S2) and Sanger DNA sequencing (Figure 2A) verified that only strains bearing *sif285-1* carry the *sui3-E189K* mutation.

We performed a series of experiments to confirm that the slow-growth-suppression and Gcn^–^ phenotypes of *sif285-1* were allelic with an E189K mutation in *SUI3*. Firstly, both *CEN* and two micron plasmids bearing WT *SUI3* were transformed into WT and *sif285-1* strains. The fast growth rate of *sif285-1 gcd6-F250L* double mutant suppressor strains is complemented by additional copies of *SUI3*. Thus slower growth is restored to *sif285-1* cells bearing *gcd6-F250L* and *SUI3* plasmids (Figure 2B; compare rows 8 and 12 with row 4), as expected if *sif285-1* is a mutation in *SUI3*. Secondly, site-directed mutagenesis introduced *sui3*-E189K onto a plasmid that was shuffled into a ‘clean’ *sui3*Δ strain as the sole source of eIF2β. The resulting strain grew as WT, indicating that like *sif285-1, sui3-E189K* does not confer a significant growth defect (Figure 2C). Thirdly, examining GAAC in these strains revealed that *sui3-E189K* confers a Gcn^–^ phenotype and fails to derepress *HIS4-lacZ* expression in response to 3AT treatment (Figure 2C) analogous to the *sif285-1* results shown in Figure 1F. Finally we created a *gcd6Δ sui3Δ* double knock out strain and complemented each deletion with either WT or mutant versions of each gene. These strains re-created the original slow-growth phenotype of *gcd6-F250L* and its suppression by *sui3-E189K* (compare Figure 2D with Figure 1A). Taken together, these data are fully consistent with *sui3-E189K* being the cause of the *sif285-1* suppressor phenotype.

### *sui3*-E189 conservation

**Figure 3.**
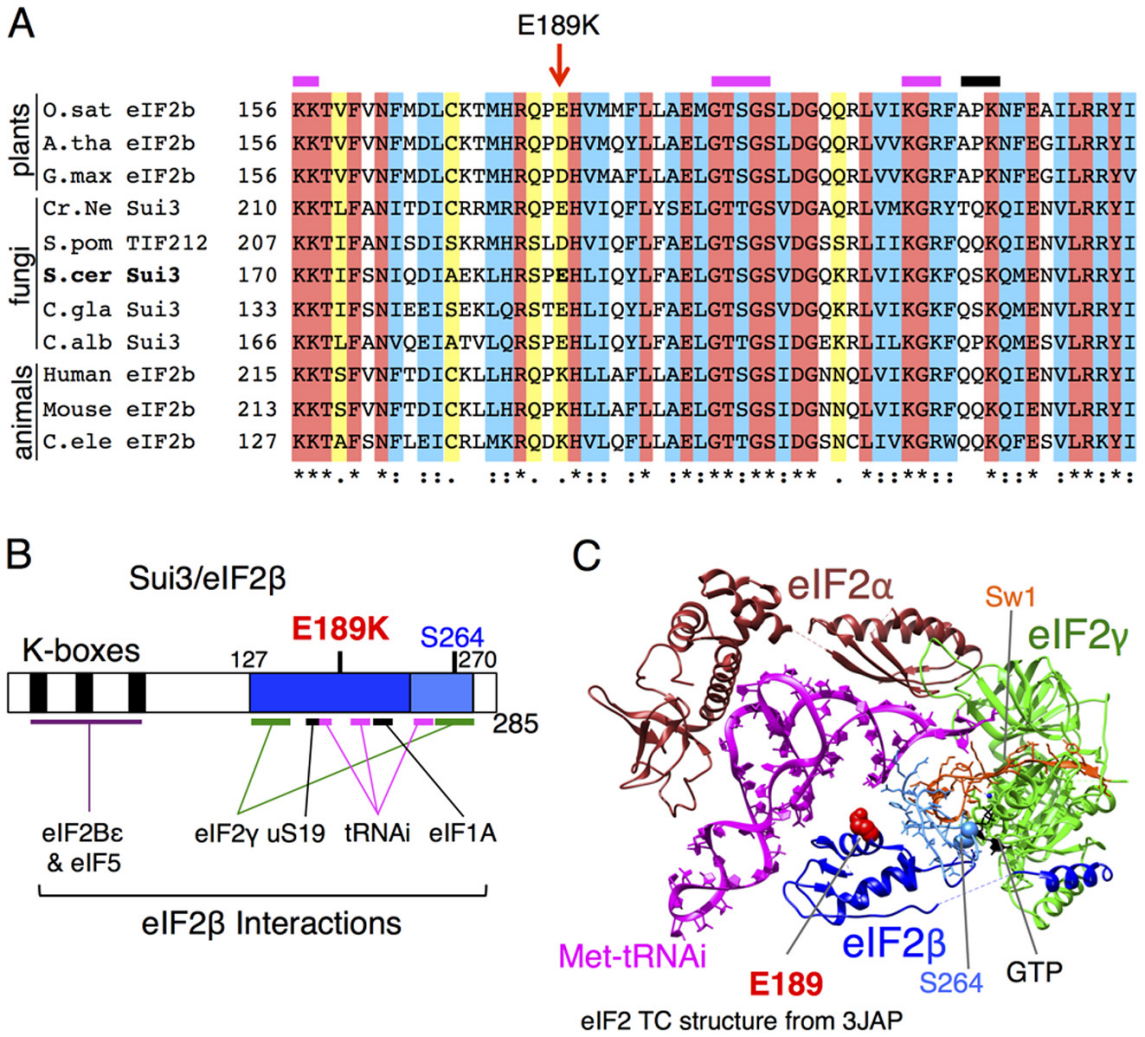
E189 is conserved in eIF2β. Please view the online version for a colour version of this figure. (**A**) Multiple sequence alignment (Clustal X) of eIF2β proteins from diverse eukaryotes showing homology around residue E189. Red highlight, identical, blue and yellow show similarity. Accession numbers for sequences are given in the ‘Materials and Methods’ section. (**B**) Cartoon with relative position of mutated E189 residue in Sui3/eIF2β protein, as well as the S2264Y (*SUI3-*2) mutation and conserved N-terminal lysine boxes described in previous studies and referred to in the text. The region with determined molecular structure is shown (blue). (**C**) Model of yeast eIF2 ternary complex structure showing Met–tRNA_i_ (magenta) and GTP (black) binding to eIF2 (brown-α, green-γ and blue-β). Residues E189 (red) and S264 (light blue) are shown filled in eIF2β. The C-terminus of eIF2β is shown in light blue. It binds both initiator Met–tRNA_i_ and Switch 1 (gold) of eIF2γ. The image was generated using UCSF Chimera software and the protein data bank coordinate file 3JAP. Only chains 1, j, k and l are shown. GTP is the analogue phosphomethylphosphonic acid guanylate ester.

The region of eIF2β around E189 is highly conserved across diverse eukaryotes (Figure 3A). E189 itself is acidic (E or D) in plants and lower eukaryotes, while the equivalent residue is already a lysine in animal sequences including humans. The significance of this evolutionary difference is not clear. E189 is located away from mutations studied previously that exhibit different phenotypes and interaction defects (Figure 3B). The eIF2β amino-terminal poly-lysine blocks (termed ‘K-boxes’) are important for stable interactions with both eIF5 and eIF2Bϵ (26). Nuclear magnetic resonance studies showed K-box 2 binds directly to the eIF5 carboxy-terminal domain (CTD) (46). The eIF2β-CTD residue S264 has been implicated in stabilizing GTP binding and regulating GTP hydrolysis as S264Y (*SUI3-2*) permits enhanced initiation at a non-AUG start codons (47).

Recent structural analysis of the yeast PIC included structural determination for eIF2β 127-270 as part of eIF2•GTP•Met–tRNA_i_, complex bound with other initiation factors and a short AUG containing mRNA to the 40S ribosome (48). In this model the eIF2β E189 side chain projects from the surface of eIF2β, permitting potential ligand modulation. In the structural model, conserved residues close to eIF2β E189 appear to stabilize Met–tRNA_i_ binding to eIF2•GTP, while residues 218-220 contact eIF1A and ribosomal protein uS19 interacts with residues 166-169 at a different eIF2β surface. These data indicate that eIF2β makes multiple important contacts within the PIC (48) and the relative positioning of E189 suggests that E189K could alter ligand binding to eIF2 (Figure 3C). We therefore decided to perform a series of experiments to determine the biochemical impact of E189K on eIF2 ligand interactions.

### E189K does not affect GDP, GTP or tRNA affinity for eIF2

**Figure 4.**
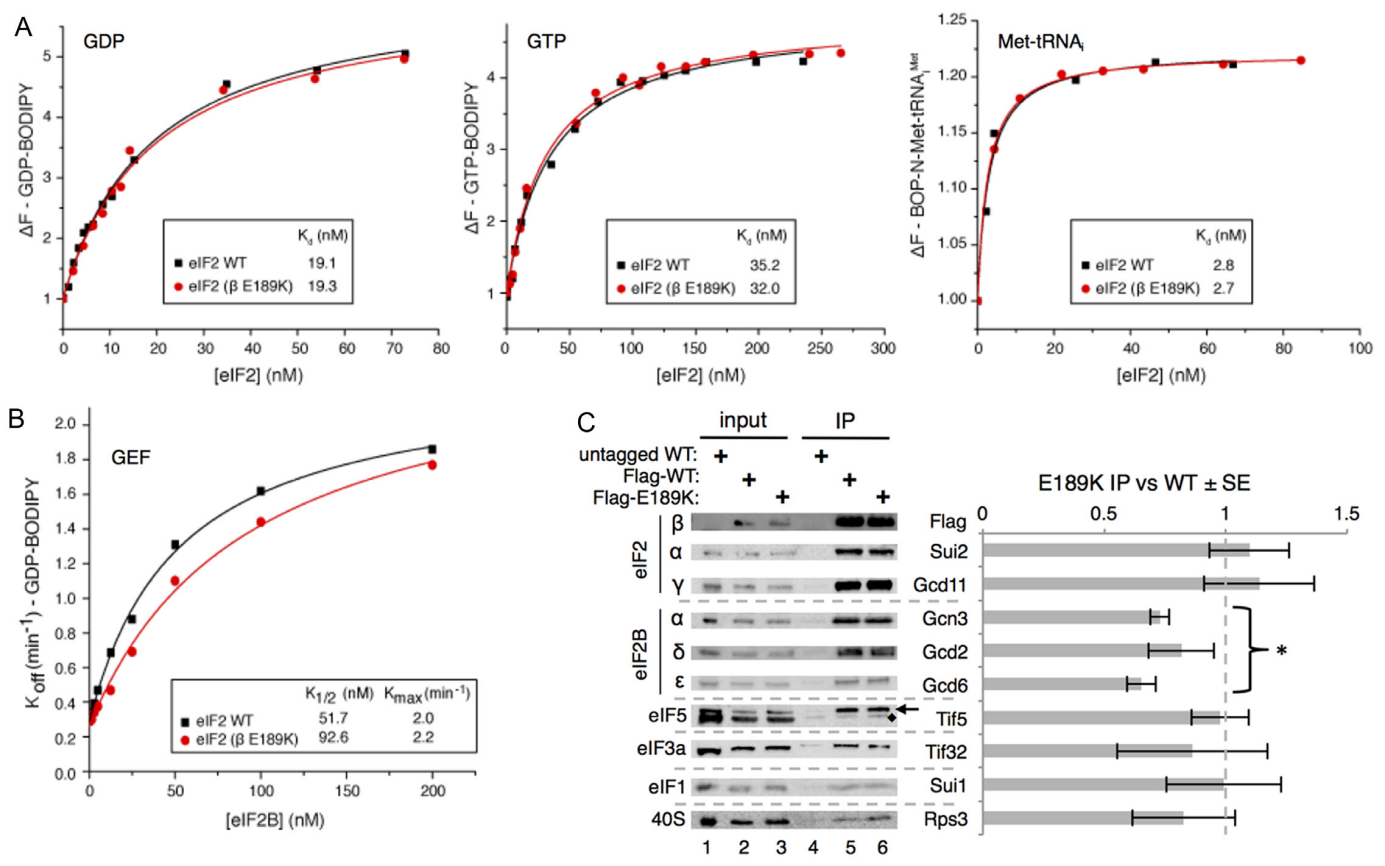
E189K has a modest impact on eIF2–eIF2B interaction and activity. (**A**) Affinity (*K*_d_) of GDP, GTP and Met–tRNA_i_ to purified WT and mutant (β E189K) apo–eIF2 complexes measured by monitoring the fluorescence intensity of 100 nM BODIPY-FL-GDP (left), 100 nM BODIPY-FL-GTP (middle) or 20 nM BOP-N-Met–tRNA_i_ with 1 mM GTP (right). (**B**) Kinetics of BODIPY-FL-GDP release from preformed eIF2 complexes in the presence of different eIF2B concentrations. *K*_1/2_ and *K*_max_ values were determined from curve fitting y = [(*K*_max_ × x)/(*K*_1/2_ + x)] + c. (**C**) Western blotting of IP of Flag-eIF2β, Flag-E189K and an untagged control from cells showing its co-association with known binding proteins. Quantification of at least three repeats using Li-Cor fluorescent secondary antibodies ±SE. Student's *t*-test indicates significant reduction in eIF2B–eIF2 interactions (*P =* 2.9 × 10^−6^) with E189K (marked *). Other factors are not significantly altered. Tif5 is indicated with an arrow, ‘♦’ marks a non-specific band.

WT and mutant eIF2 (eIF2^βE189K^) were purified from an overexpression yeast strain engineered so that the introduced plasmid was the sole source of eIF2β (Supplementary Table S1 and Figure S3A). During purification incubation with EDTA was used to remove residual bound magnesium and nucleotide, as described previously (3). To assess nucleotide and Met–tRNA_i_ binding affinities we monitored the change in fluorescence upon binding of eIF2 to ligands labelled with BODIPY derivatives at room temperature (Figure 4A). These experiments show that BODIPY-GDP and BOP-N-Met–tRNA_i_ affinities for WT eIF2 are within the ranges measured previously by other techniques (*K*_d GDP_ = 19.1 nM and *K*_d Met–tRNAi_ = 2.8 nM) (3,49). Measured BODIPY-GTP affinity is tighter than when GTP-binding was measured by filter-binding assays (3,49), so that the difference between GDP and GTP affinities is significantly less than noted previously. Perhaps surprisingly, the affinities for each ligand are unchanged by the βE189K mutation (Figure 4A). Next, we pre-bound BODIPY-GDP to eIF2 or eIF2^βE189K^, and purified these complexes away from unbound BODIPY-GDP. By incubating each complex with excess unlabelled GDP, we monitored the release of BODIPY-GDP and determined its off-rate. The *K*_off_ BODIPY-GDP was identical for both WT and eIF2^βE189K^ (WT *K*_off_ = 0.292 ± 0.025 min^−1^ and eIF2^βE189K^ *K*_off_ = 0.293 ± 0.015 min^−1^; Figure 4B, 0 nM eIF2B). These results demonstrate that eIF2β-E189K mutation does not simply alter the binding of either nucleotide or Met–tRNA_i_. Hence eIF2^βE189K^ does not share defects in ligand interactions described previously for other eIF2 mutations (47,49).

### E189K modestly reduces eIF2B GEF activity, but not PIC interactions

Because the E189K mutation suppresses the slow-growth of eIF2B mutants with reduced GEF activity, one possible mechanism of mutant action was the enhancement of eIF2B activity. We tested this idea by adding increasing concentrations of purified eIF2B (39) to purified eIF2•BODIPY-GDP to determine *K*_$\frac{1}{2}$_ and *K*_max_ values for eIF2B GEF. The measured *K*_max_ for WT eIF2 was ∼14-fold faster than the rate determined previously with a filter-binding assay (14). This likely reflects that these fluorescent assays were performed at room temperature rather than 0°C used previously. Surprisingly, the rate of nucleotide exchange from eIF2^βE189K^ complexes was modestly impaired relative to that with WT eIF2, increasing *K*_$\frac{1}{2}$_ from 51.7 to 92.6 nM eIF2B (Figure 4B). This experiment identified the first clear difference between WT and eIF2^βE189K^ complexes, but because impairment of eIF2B function by E189K should exacerbate rather than suppress the growth defect of eIF2B mutants, this observation cannot readily account for the growth suppression phenotype uncovered.

As outlined above, eIF2 contacts other ligands within the PIC including eIF5, 40S ribosomes, eIF3 and eIF1. To examine eIF2–protein interactions more widely, we performed anti-FLAG immunoprecipitation of FLAG-eIF2β from WT and E189K cells and assessed the ability of known eIF2-interacting partners to co-associate by quantitative western blotting. The only significant defect observed was a small, but statistically significant, 20% reduction in association of eIF2B subunits with eIF2β-E189K observed upon quantification of the signals (Figure 4C). Together with the modest reduction in activity seen in the GEF assays, these results suggest that one consequence of the eIF2β mutation is impaired eIF2B binding and activity. In contrast, binding of eIF2 to PIC factors eIF5, eIF3, eIF1 and 40S ribosomes appears unchanged (Figure 4C), consistent with the growth characteristics of eIF2β-E189K cells. These data support the idea that the E189K mutation does not significantly, if at all, interfere with eIF2 functions in PIC formation, and scanning/AUG recognition, but does modestly reduce eIF2B interaction and activity.

### eIF2β-E189K eliminates eIF5 GDI activity

**Figure 5.**
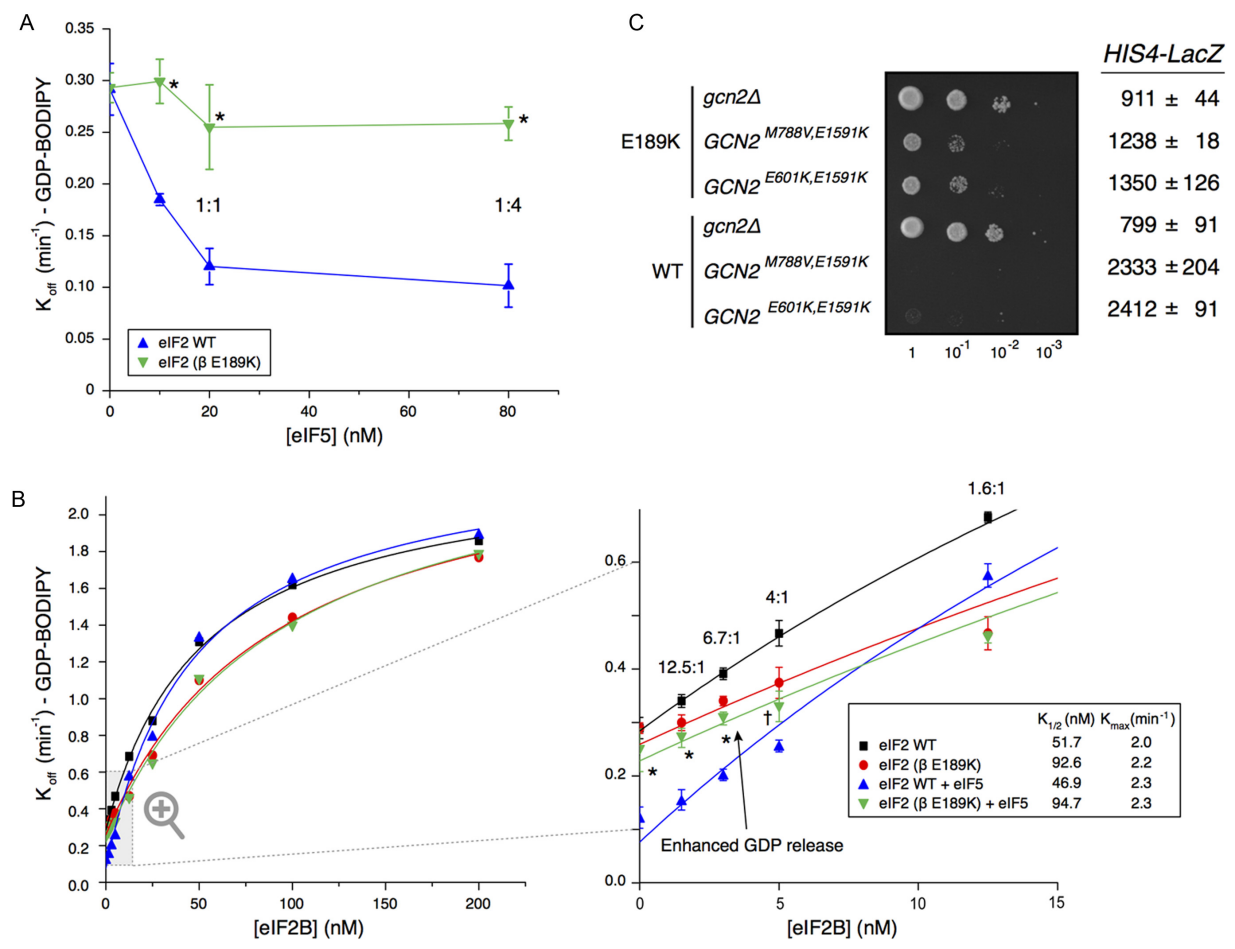
E189K antagonizes eIF5 GDI activity. (**A**) Kinetics of BODIPY-FL-GDP release from preformed purified WT and mutant (β E189K) eIF2•GDP complexes (20 nM) with varying concentrations of GST-eIF5 ± SD (*n* = 3). Molar eIF2:GST–eIF5 protein ratios are shown. Asterisks (*) mark points with statistically significant difference to WT (*P* < 0.01, unpaired Student's *t*-test). (**B**) Kinetics of BODIPY-FL-GDP release from preformed eIF2 complexes (20 nM) in the presence of different eIF2B concentrations and eIF5 (20 nM). Curves shown in Figure 3B are repeated for direct comparison. Right panel shows a zoomed image focussing on eIF2B 0–12.5 nM ± SD (*n* = 3). Statistical significant increase in the rate of GDP release from eIF5/eIF2^βE189K^ relative to eIF5/eIF2 WT is indicated (* = *P* < 0.01, † = *P* < 0.05, unpaired Student's *t*-test). (**C**) Growth and *HIS4-LacZ* expression of E189K and WT eIF2 cells (strains GP3773 and GP3771) transformed with plasmids carrying the indicated constitutively active *GCN2* alleles.

We found previously that eIF5 mutants that impair its GDI activity can partially suppress slow-growth phenotypes of eIF2Bϵ mutations and confer a Gcn^–^ phenotype (14,23) in a manner similar to that seen with eIF2^βE189K^ and eIF2B alleles, including rescue of the lethality of the *gcd6-N249K* mutation (Supplementary Figure S1C). This suggests a link between eIF2^βE189K^ and eIF5 GDI mutants. However, the eIF5 GDI mutants characterized act, at least in part, by reducing the affinity of eIF5 for eIF2 (23), whereas the eIF2^βE189K^ mutation does not reduce eIF5 interaction *in vivo* (Figure 4C) or *in vitro* (Supplementary Figure S3B). These observations imply that reduced eIF5 binding to eIF2 does not explain the suppressor phenotype. We therefore decided to assess eIF5 GDI activity directly. We monitored eIF5 GDI activity by incubating eIF2•BODIPY-GDP (20 nM) with purified eIF5 (0–80 nM) and recorded the fluorescence change upon dissociation of BODIPY-GDP from eIF2. GDP-binding to WT eIF2 was stabilized by eIF5 in line with previous observations implying that a 1:1 eIF2•GDP/eIF5 complex forms that stabilizes GDP binding to eIF2 (23). Importantly GDP binding to eIF2^βE189K^ was not stabilized by eIF5 even at excess eIF5 levels (Figure 5A). Hence the experiment shows that eIF2^βE189K^ eliminates eIF5 GDI activity and this finding represents the likely cause contributing to the original *gcd6-F250L* slow-growth suppression phenotype observed in *sif285-1*/sui3-E189K mutant cells (Figure 1).

To examine the impact of eIF5 on eIF2B GDF and GEF activities, we monitored BODIPY-GDP release from eIF2 pre-bound to eIF5 over a wide range of eIF2B concentrations. In line with previous observations (14) GDP release from WT eIF2 was inhibited at low eIF2B concentrations by eIF5 GDI. At higher eIF2B levels GDP release is unaffected by eIF5 because excess eIF2B can fully displace eIF5, nullifying GDI (compare blue [+eIF5] with black [–eIF5] in Figure 5B, left panel). In contrast, even at low eIF2B concentrations the kinetics of GDP-release from eIF2^βE189K^ are completely unaffected by the presence of eIF5 (compare green with red symbols in Figure 5B, zoomed right panel).

eIF2 is between 6-12–fold in excess over eIF2B *in vivo* (22,50). When eIF2B is mutated or eIF2α phosphorylated at serine 51, eIF2B activity is reduced such that it is limiting for translation initiation (14,41). In our assays at low levels of active eIF2B (below 8 nM eIF2B in Figure 5B, zoomed right panel) the rate of GDP release from eIF5/eIF2^βE189K^ is accelerated relative to eIF5/eIF2 WT (Figure 5B, right panel, compare green and blue symbols). Our assays contained 20 nM each of eIF2•GDP and eIF5, so eIF2B concentrations below 8 nM likely reflect factor ratios *in vivo*. The increased rate of GDP release from eIF2^βE189K^ observed with 5 nM or less eIF2B is statistically significant (points marked * or † in Figure 5B, right panel) and is fully consistent with the slow-growth suppression phenotype observed in cells where low eIF2B GEF activity normally limits growth-rate (Figures 1 and 2). To conclude, our data indicate that eIF2β-E189K eliminates eIF5 GDI activity and thereby facilitates faster rates of nucleotide exchange when eIF2B activity is limiting. This is consistent with our observations of enhanced protein synthesis rates and enhanced rates of cell growth in eIF2β-E189K/*sif285-1* cells bearing defective eIF2B mutants with lower eIF2B GEF activity.

Physiologically eIF2B activity is reduced *in vivo* by phosphorylation of eIF2α at serine 51. A prediction of our genetic (Gcn^–^ phenotype) and biochemical results (loss of GDI activity) is that eIF2β-E189K should reduce the sensitivity of cells to very high levels of phosphorylated eIF2. We tested this hypothesis by transforming cells with plasmids expressing one of two constitutively-active alleles of the Gcn2 kinase (*GCN2^C^*) that each cause high levels of eIF2(αP) and result in severe slow-growth (51). In accord with our predictions, eIF2β-E189K reverted the slow-growth of both *GCN2^C^* mutants tested and lowered the aberrantly high expression of the *HIS4-LacZ* reporter seen in *SUI3 GCN2^C^*cells (Figure 5C). Our data demonstrate that eIF2β–E189 and GDI are critical for the normal tight control of translation and GAAC by eIF2(αP).

## DISCUSSION

In this study, we have described the serendipitous discovery and subsequent genetic identification and molecular analysis of a growth suppressor mutation that spontaneously arose during our studies of eIF2B GEF mutants. *sif285-1* is a novel missense allele within the β subunit of eIF2 (E189K) that does not affect the intrinsic nucleotide, Met–tRNA_i_ or 43S PIC-binding affinities of eIF2. Instead we find that it prevents eIF5 GDI activity. This single amino acid change alters the sensitivity of cells to reduced eIF2B activity thereby repressing the cellular GAAC response to elevated eIF2(αP). Taken together our data show that eIF2β is critically important for guiding the GDI activity of eIF5. They also demonstrate that eIF2β is important for determining the normal cellular responses to eIF2(αP) including activation of *GCN4* target genes during GAAC.

*sui3-E189K* is the first eIF2 mutation described that prevents stabilization of GDP binding to eIF2γ by eIF5 GDI. Previously described mutations in eIF2 subunits typically have different defects in protein synthesis. For example mutations in eIF2γ that directly alter its affinity for GTP/GDP or Met–tRNA_i_ (49); Sui^−^ mutations in each of the three eIF2 subunits that enhance initiation at non-AUG codons (47,52–54); mutations that affect subunit interactions and impair eIF2 complex integrity (55). All these mutations impair cell growth and protein synthesis. In addition, the three repeated lysine blocks (K-boxes) within the amino terminus of eIF2β have been found critical for binding to both eIF5 and eIF2Bϵ and their complete deletion is lethal *in vivo* (26,46,56). *sui3-E189K* shares growth properties with Gcn^–^ mutations in eIF2α that alter the ability of eIF2 to respond to stress/GAAC signalling. These alleles were shown to overcome the inhibition of translation by phosphorylated eIF2 by either impairing interactions between eIF2α and eIF2 kinases or by weakening the tight binding of phosphorylated eIF2 to eIF2B (57,58). Gcn^–^ mutations have also been found in other PIC factors including eIF1A (59), eIF3 subunits (60) and eIF5 (61). *GCN4* reporter gene analyses of these Gcn^–^ mutations revealed defects in uORF1 AUG codon recognition (leaky-scanning) or scanning contribute to the Gcn^–^ phenotypes. However these mutants also cause general defects in protein synthesis leading to aberrant polysome profiles and/or poor rates of growth, presumably caused by widespread initiation defects in one or more of the steps in protein synthesis initiation on many mRNAs. *sui3-E189K* does not share these global translation defects (Figures 1 and 2) and in contrast improves growth of otherwise sick eIF2B mutant strains. In addition our biochemical analyses (Figure 4) indicate that the E189K mutation does not significantly, if at all, interfere with eIF2 interactions with PIC factors. This is again in contrast to similar analyses with mutants that do interfere with functions in PIC formation, scanning or AUG recognition (59–61). Hence *sui3-E189K* is the first Gcn^–^ mutant in eIF2β and defines a novel class of eIF2 mutation that impairs eIF5 GDI function (Figure 5A).

Previously we studied features of eIF5 necessary for GDI activity. We found that GDI requires the eIF5-CTD that interacts with eIF2β, as well as the eIF5 LR containing a conserved DWEAR motif that binds to eIF2γ (23). Mutations within the eIF5-CTD and DWEAR motif were found to weaken eIF5 binding to eIF2 and to eliminate GDI (23). Those data suggested that eIF5 GDI was functioning primarily via protein–protein interactions with eIF2β and γ. As eIF2γ binds GDP we suggested that it was eIF5–eIF2γ interactions that were most critical for GDI. However, our new data imply that eIF2β has a more critical role in mediating eIF5 GDI, and does more than simply providing an anchoring point for eIF5 through eIF5-CTD–eIF2β K-box interactions, because eIF5 binds to eIF2^βE189K^ as well as it does to WT eIF2 (Figure 4C and Supplementary Figure S3B), but has no GDI activity (Figure 5A).

Recent cryo-electron microscopy of the yeast PIC of eIF2 within the PIC has provided significant insight into the interactions between eIF2 subunits, GTP, Met–tRNA_i_ and other PIC proteins and 40S ribosomes. However, density associated with eIF5 was not defined (48). As shown in Figure 3C, the structure indicates that eIF2β-E189 sits at the surface of eIF2β with its side-chain projecting into the solvent and is close to residues that interact with Met–tRNA_i_. In contrast eIF2β–S264, a residue implicated in controlling GTP-hydrolysis is within the CTD (pale blue in Figure 3C). The eIF2β-S264Y mutation (*SUI3-2*) promotes premature hydrolysis of eIF2-bound GTP, permitting aberrant initiation at non-AUG codons (47). The EM structure shows that eIF2β-S264 binds both the tRNA_i_ acceptor arm and the eIF2γ GTP-binding domain including the conserved switch 1 region that is important for controlling nucleotide binding (Sw1, orange in Figure 3C). S264 itself is juxtaposed to eIF2γ-bound GTP and is therefore ideally placed to limit nucleotide release from eIF2γ (48). There may be mechanistic similarities between eIF2β's role in the PIC and its role in the eIF2•GDP/eIF5 complex. Within the PIC in WT cells eIF2β-S264 is critical to prevent premature hydrolysis of GTP and Pi release by eIF5 at non-AUG codons. Here we now show that in eIF2•GDP/eIF5 complexes, eIF2β it is critical for eIF5 GDI to prevent premature eIF2B-independent release of GDP (47). Thus in both reactions eIF2β is important for ensuring tight regulation of eIF2 nucleotide status.

**Figure 6.**
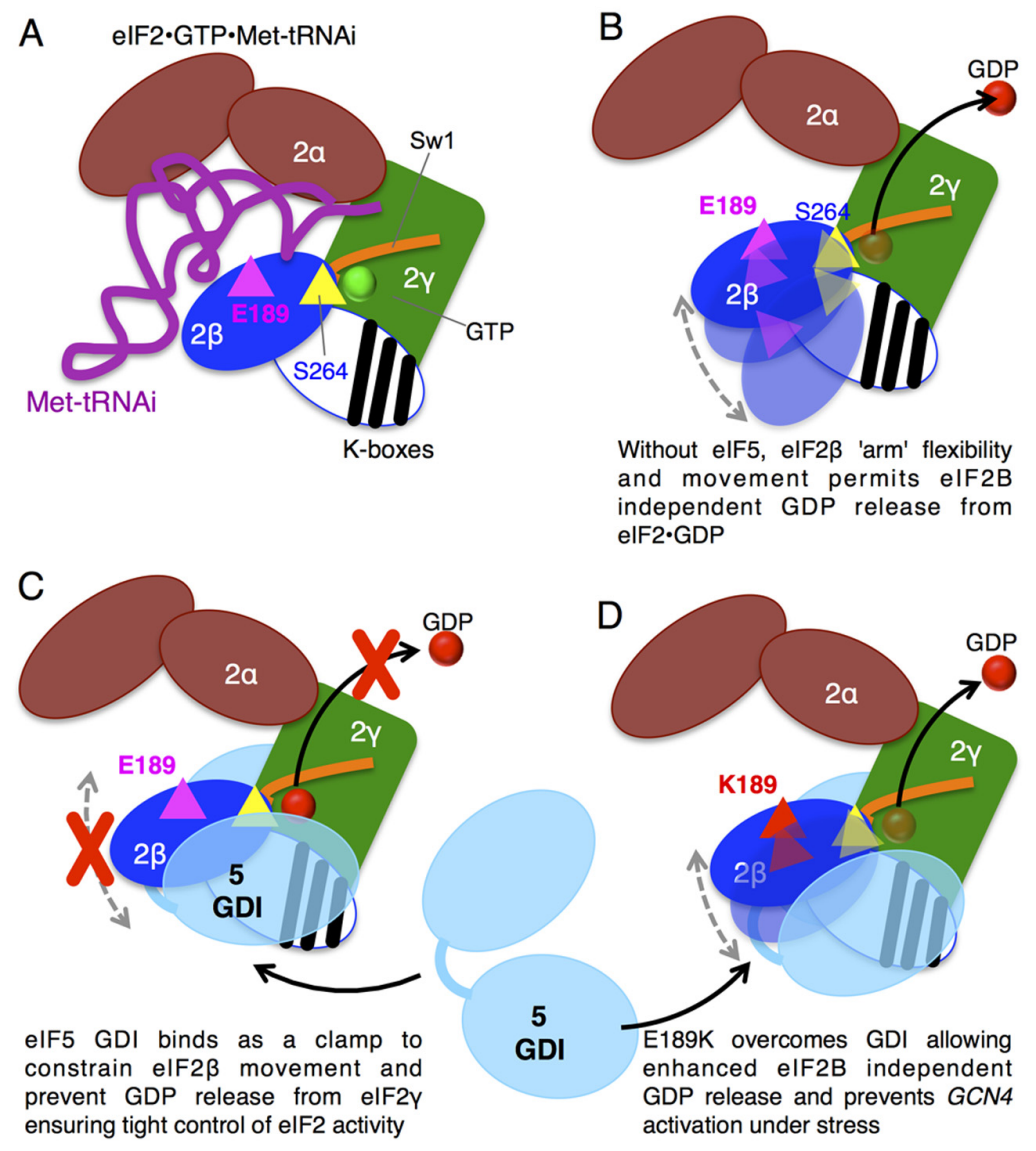
Model for role of βE189 ensuring tight control of GDP release from eIF2. (**A**) eIF2 heterotrimer is shown in cartoon form based on the structure model shown in Figure 3C with eIF2γ core bound to GTP and with α and β ‘arms’ ‘grasping’ Met–tRNA_i_ (purple). The structure of the N-terminal region of eIF2β bearing the lysine repeat ‘K-boxes’ is not known and is shown here as a white oval with black K-box stripes and is speculatively positioned over eIF2γ. βS264 is depicted as a yellow triangle adjacent to GTP bound to eIF2γ G domain and Sw1 and βE189 is shown as a magenta triangle. (**B**) Following GTP hydrolysis and eIF2 release from the PIC and in the absence of other ligands, eIF2β ‘arm’ position is flexible and its movement contributes to the relatively high off-rate of GDP. (**C**) Binding of eIF5 GDI acts as a molecular clamp constraining eIF2β and stabilizing GDP binding to eIF2γ. (**D**) E189K alters eIF5-eIF2 interactions so that GDP is no longer stabilized despite eIF5 interaction, shown by eIF2β ‘arm’ flexibility.

eIF5 is composed of two structured domains joined by an LR (62). There are separate structures for the amino terminal GAP domain and a CTD. The CTD is α-helical forming HEAT repeats that are shared with the GEF domain of eIF2Bϵ (63,64) and region of the CTD is implicated in binding to the K-boxes of eIF2β (65) (46). The DWEAR motif implicated in eIF5 GDI lies just upstream of the crystallized CTD (63), so was placed in the LR (23). However, structure predictions indicate it is likely also α-helical and is probably an extended part of the CTD, similar to the longer α-helical CTD of related eIF5 mimic proteins (66). Combining the known structural and new functional information provides a framework for developing a model for eIF5 GDI function. This is shown in cartoon form in Figure 6, with elements of eIF2 depicted to mimic the structure view shown in Figure 3C. From the PIC structure, eIF2 has a core gamma subunit with eIF2α and β acting as ‘arms’ constrained by interactions with Met–tRNA_i_ (Figure 6A). Upon GTP hydrolysis and release of Met–tRNA_i_, eIF2•GDP is released from the initiation complex and likely retains a similar overall form, but because the subunits are no longer constrained by Met–tRNA_i_ interactions, the α and β arms may enjoy enhanced conformational flexibility. eIF2β movement relative to eIF2γ could permit the GDP release we observe in the absence of other ligands. This idea is shown in Figure 6B. We propose that eIF5 binding to eIF2β and γ acts as a ‘molecular clamp’ to stabilize an eIF2 conformation that prohibits GDP release (Figure 6C). Hence eIF5 GDI ensures tight eIF2B-dependent regulation of eIF2•GDP/GTP cycling. In contrast, when eIF5 binds to eIF2^βE189K^ it cannot stabilize the conformation needed to prevent low-level GDP release (Figure 6D) and thereby enables faster growth to eIF2B mutants with low GEF activity (Figures 1 and 2) and prevents tight regulation of translation and GAAC by eIF2(αP) (Figures 1D, 2 and 5C). To test this hypothetical model will likely require significant progress toward understanding structures and dynamics of the interactions between eIF5 and the eIF2 complex, which is beyond the scope of this study.

## Supplementary Material

SUPPLEMENTARY DATA
