# Supplementary material for: eIF2β is critical for eIF5-mediated GDP-dissociation inhibitor activity and translational control
Source: Nucleic Acids Res. 2016 Jul 25;44(20):9698–709. doi: 10.1093/nar/gkw657 (PMC5175340; doi:10.1093/nar/gkw657)
Supplement: SUPPLEMENTARY DATA [file supp_44_20_9698__index.html]

eIF2β is critical for eIF5-mediated GDP-dissociation inhibitor activity and translational control — SUPPLEMENTARY DATA 

# eIF2β is critical for eIF5-mediated GDP-dissociation inhibitor activity and translational control

## SUPPLEMENTARY DATA

- SUPPLEMENTARY DATA
