# Supplementary material for: eIF2β is critical for eIF5-mediated GDP-dissociation inhibitor activity and translational control
Source: Nucleic Acids Res. 2016 Jul 25;44(20):9698–709. doi: 10.1093/nar/gkw657 (PMC5175340; doi:10.1093/nar/gkw657)

Supplementary Materials for

eIF2β is critical for eIF5-mediated GDP-dissociation inhibitor activity and translational control

Martin D. Jennings, Christopher J. Kershaw, Christopher White, Danielle Hoyle, Jonathan P. Richardson, Joseph L. Costello, Ian J. Donaldson, Yu Zhou and Graham D. Pavitt

Contents

Supplementary Table S1……………………………………………………………………….p2

Supplementary Table S2……………………………………………………………………….p3

Supplementary Table S3……………………………………………………………………….p4

Supplementary References…………………………………………………………………….p5

Supplementary Figure Legends……………………………………………………………….p6

Supplemental Figure 1…………………………………………………………………….…..p7

Supplemental Figure 2…………………………………………………………………….…..p8

Supplemental Figure 3…………………………………………………………………….…..p9

Supplementary Tables

Supplementary Table S1. Main strains used[1]

Stain	Genotype	Source
GP3755	*MAT*α *leu2-3 leu2-112 ino1 gcd6*Δ *gcn2*Δ::*hisG ura3-52*::*P_{HIS4}-lacZ* pAV1513[*GCD6 CEN URA3*]	(1)
GP3756	*MAT*α *leu2-3 leu2-112 ino1 gcd6*Δ *gcn2*Δ::*hisG ura3-52*::*P_{HIS4}-lacZ* pAV1524[*gcd6-F250L CEN URA3*]	(1)
GP3763	GP3756: *sif285-1*(*sui3-E189K*)	*This study, spontaneous slow-growth suppressor*
GP3771	GP3755: pAV1265 [*GCD6 CEN LEU2*]	*This study, plasmid shuffle*
GP3772	GP3755: pAV1592 [*gcd6-F250L CEN LEU2*]	*This study, plasmid shuffle*
GP3773	GP3763: pAV1265 [*GCD6 CEN LEU2*]	*This study, plasmid shuffle*
GP3774	GP3763: pAV1592 [*gcd6-F250L CEN LEU2*]	*This study, plasmid shuffle*
GP3779	GP3771: *MAT***a**	*This study, HO mating switch, FOA, MAT***a** *spore*
GP3785	GP3773: *MAT***a**	*This study, HO mating switch, FOA, MAT***a** *spore*
GP3932	*MAT***a**/α *leu2-3/leu2-3 leu2-112/leu2-112 ura3-52*::*P_{HIS4}-lacZ/ura3-52*::*P_{HIS4}-lacZ ino1/ ino1 gcd6*Δ*/gcd6*Δ *gcn2*Δ::*hisG/gcn2*Δ::*hisG SIF285* (*SUI3*)/*sif285-1*(*sui3-E189K*) pAV1524[*gcd6-F250L URA3 CEN*]	*This study, diploid strain heterozygous for sif285-1*
GP4146	*MAT*α*/MAT***a** *his3-*Δ*1/his3-*Δ*1 leu2*Δ*0/leu2*Δ*0 met15*Δ*0/MET15 LYS2/lys2*Δ*0; ura3*Δ*0/ura3*Δ*0 GCD6/ gcd6*Δ::KanMX4	*Euroscarf Y23570*
GP4907	*MAT*α *gcn2*Δ *ino1 leu2-3 leu2-112 sui3*Δ *ura3-52*::*P_{HIS4}-lacZ* p920 [*SUI3 LEU2*]	*K. Asano Kansas Sate University strain KAY17*
GP5010	*MAT*α *gcn2*Δ *ino1 leu2-3 leu2-112 sui3*Δ *trp1*Δ::*hisG ura3-52*::*P_{HIS4}-lacZ* p920 [*SUI3 LEU2*]	(2)
GP5949	*MAT*α *prb1-1122 pep4-3 leu2 trp1 ura3-52 gal2* pAV1353 [*GCN3 GCD2 GCD7 URA3* 2μm] pAV1413 [*GCD1*-FLAG2-His6 *GCD6 LEU2* 2μm]	(3)
GP7124	GP5010: pAV2443 [Flag-*SUI3 TRP1*]	*This study, plasmid shuffle in GP5010*
GP7125	GP5010: pAV2452 [Flag-*SUI3 E189K TRP1*]	*This study, plasmid shuffle in GP5010*
GP7145	*MAT*α *leu2-3 leu2-112 ino1 sui3*Δ *gcn2*Δ *ura3-52*::*P_{HIS4}-lacZ* pAV2451 [*SUI2* V5-*SUI3-E189K* His6-*GCD11 LEU2* 2μm]	*This study, plasmid shuffle in GP4907/KAY17*
GP7146	*MAT*α *leu2-3 leu2-112 ino1 sui3*Δ *gcn2*Δ *ura3-52*::*P_{HIS4}-lacZ*. pAV2322 [*SUI2* V5-*SUI3* His6-*GCD11 LEU2* 2μm]	*This study, plasmid shuffle in GP4907/KAY17*
GP7216	*MAT*α *gcd6*Δ::KanMX *gcn2*Δ *leu2-3 leu2-112 ino1 sui3*Δ *trp1*Δ::*hisG ura3-52*::*P_{HIS4}-LacZ*. pAV2444 [Flag-*SUI3 LEU2*] pAV1513 [*GCD6 URA3 CEN*]	*This study, knockout of GCD6 by PCR of gcd6*Δ*::KanMX from GP4146 in a GP5010 derived strain.*
GP7217	As GP7216, but with pAV1524 [*gcd6-F250L URA3 CEN*]	*This study, as GP7216*
GP7218	As GP7216, but with pAV2452 [Flag-*SUI3 E189K TRP1*]	*This study, as GP7216*
GP7219	As GP7217, but with pAV2452 [Flag-*SUI3 E189K TRP1*]	*This study, as GP7216*

[1]For clarity, additional diploid strains (see Supplementary Figure 1D) or strains derived from those above by transformation with *GCN2* or vector plasmids are not indicated here.

Supplementary Table S2. Plasmids Used.

Name	Yeast Genes	Source/ Reference
pAV1195/ pRS316	*URA3 CEN*	(4)
pAV1198/ p722	*GCN2 URA3 CEN*	(6)
pAV1229/ p921	*SUI3 URA3 CEN*	(5)
pAV1233/ p927	*SUI3 URA3* 2µm	(5)
pAV1248/ p1055	*GCN2-M788V,E1591K URA3 CEN*	(6)
pAV1249/ p1056	*GCN2-E601K,E1591K URA3 CEN*	(6)
pAV1265	*GCD6 CEN LEU2*	(7)
pAV1353	*GCN3 GCD2 GCD7 URA3* 2µm	(3)
pAV1413	*GCD1*-FLAG2-His6 *GCD6 LEU2* 2µm	(3)
pAV1427	P_{GAL1}-*GCD6 leu2-d URA3* 2µm	(1)
pAV1456	P_{GAL1}-*gcd6-F250L leu2-d URA3* 2µm	(1)
pAV1513	*GCD6 CEN URA3*	(1)
pAV1516	*gcd6-N249K CEN URA3*	(1)
pAV1524	*gcd6-F250L CEN URA3*	(1)
pAV1586	*gcd6-S576N CEN URA3*	(1)
pAV1582	*gcd6-T552I CEN URA3*	(1)
pAV1592	*gcd6-F250L CEN LEU2*	(1)
pAV1641/ pHO1.5	*HO URA3* 2µm	MJR Stark, Dundee University
pAV1900	GST-*TIF5* (*E. coli* expression vector pGEX-4T1)	(8)
pAV2322	*SUI2* V5-*SUI3* His_6-*GCD11 LEU2* 2µm	*This study*. N-terminal V5-tag added to *SUI3* in pAV1726
pAV2443/ KAB258	Flag-*SUI3 TRP1 CEN*	(9)
pAV2444/ KAB502	Flag-*SUI3 LEU2 CEN*	(9)
pAV2451	*SUI2* V5-*SUI3-E189K* His6-*GCD11 LEU2* 2µm	*This study.* Site-directed mutagenesis of pAV2322
pAV2452	Flag-*SUI3-E189K TRP1 CEN*	*This study.* Site-directed mutagenesis of pAV2443

Supplementary Table S3. Candidate single nucleotide variants (SNVs) detected in *sif285-1* vs wild type cells by ABI SOLiD 4 genome sequencing

Chromosome: position	WT	Mut	read depth[1]		ORF	GENE	codon change	amino acid change
			Raw	high-quality				
chrIV: 885,474	T	C	52	50	YDR211W	*GCD6*	TTT>CTT	F250L
chrIX: 142,390	C	T	42	35	intergenic	NA	NA	NA
chrX: 672,311	C	T	108	91	yJR132w	*NMD5*	CGC>CGT	R930R[2]
chrXI: 94,945	C	A	119	83	yKL185w	*ASH1*	TCC>TCA	S149S[2]
chrXII: 688,246	C	A	100	62	intergenic	NA	NA	NA
chrXII: 734,649	A	G	141	136	intergenic	NA	NA	NA
chrXVI: 101,060	G	A	112	97	ypl237w	*SUI3*	GAA>AAA	E189K

[1] The total number of reads mapped at this genome position in the *sif285-1* strain (GP3563) and the number of high quality mutant reads

[2]Silent change not investigated further.

Supplementary References

1. Gomez, E. and Pavitt, G.D. (2000) Identification of domains and residues within the epsilon subunit of eukaryotic translation initiation factor 2B (eIF2Bepsilon) required for guanine nucleotide exchange reveals a novel activation function promoted by eIF2B complex formation. *Molecular and cellular biology*, **20**, 3965-3976.
2. de Almeida, R.A., Fogli, A., Gaillard, M., Scheper, G.C., Boesflug-Tanguy, O. and Pavitt, G.D. (2013) A yeast purification system for human translation initiation factors eIF2 and eIF2Bepsilon and their use in the diagnosis of CACH/VWM disease. *PloS one*, **8**, e53958.
3. Mohammad-Qureshi, S.S., Haddad, R., Palmer, K.S., Richardson, J.P., Gomez, E. and Pavitt, G.D. (2007) Purification of FLAG-tagged eukaryotic initiation factor 2B complexes, subcomplexes, and fragments from Saccharomyces cerevisiae. *Methods in enzymology*, **431**, 1-13.
4. Sikorski, R.S. and Hieter, P. (1989) A system of shuttle vectors and yeast host strains designed for efficient manipulation of DNA in Saccharomyces cerevisiae. *Genetics*, **122**, 19-27.
5. Dever, T.E., Yang, W., Astrom, S., Bystrom, A.S. and Hinnebusch, A.G. (1995) Modulation of $tRNA_i^{Met}$, eIF-2 and eIF-2B expression shows that *GCN4* translation is inversely coupled to the level of eIF-2·GTP·Met-$tRNA_i^{Met}$ ternary complexes. *Mol. Cell. Biol.*, **15**, 6351-6363.
6. Ramirez, M., Wek, R.C., Vazquez de Aldana, C.R., Jackson, B.M., Freeman, B. and Hinnebusch, A.G. (1992) Mutations activating the yeast eIF-2 alpha kinase GCN2: isolation of alleles altering the domain related to histidyl-tRNA synthetases. *Molecular and cellular biology*, **12**, 5801-5815.
7. Bushman, J.L., Asuru, A.I., Matts, R.L. and Hinnebusch, A.G. (1993) Evidence that GCD6 and GCD7, translational regulators of GCN4, are subunits of the guanine nucleotide exchange factor for eIF-2 in Saccharomyces cerevisiae. *Molecular and cellular biology*, **13**, 1920-1932.
8. Jennings, M.D. and Pavitt, G.D. (2010) eIF5 has GDI activity necessary for translational control by eIF2 phosphorylation. *Nature*, **465**, 378-381.
9. Asano, K., Krishnamoorthy, T., Phan, L., Pavitt, G.D. and Hinnebusch, A.G. (1999) Conserved bipartite motifs in yeast eIF5 and eIF2Bepsilon, GTPase-activating and GDP-GTP exchange factors in translation initiation, mediate binding to their common substrate eIF2. *The EMBO journal*, **18**, 1673-1688.

Supplementary Figure Legends.

Supplementary Fig. S1. Genetic characterisation of a spontaneous extragenic suppressor of eIF2Bε mutations

A. Faster growing colonies (arrowed) appear on re-streaking strain GP3756 (*gcd6*-F250L) from a petri plate stored at 4°C. **B.** Transformation and plasmid shuffling of *gcd6* alleles shows slow-growth suppressor is extragenic. Growth of strains GP3771-3774 on YPD. **C.** Transformation and plasmid shuffling of *gcd6* alleles shown into GP3771 (WT, left) and GP3773 (*sif285-1*, right). Both day 2 (top) and day 3 (bottom) growth is shown. Right, a summary table of growth phenotypes of strains shown in panels B and C. **D.** *sif285-1* is semi-dominant in diploid strains generated by crosses. Strains crossed are: GP3755 x GP3779 (1); GP3756 x GP3779 (2); GP3756 x GP3785 (3); GP3763 x GP37585 (4). Following each cross, pAV1265 was lost by 'unselected growth' so that the indicated *gcd6* allele was present on a *URA3* plasmid. **E.** Composite figure of ten independent four-spore tetrads dissected from diploid strain GP3932.

Supplementary Fig. 2. E189K mutation eliminates BspE1 site in *SUI3.*

1% agarose gel electrophoresis BspE1 digest of *SUI3* ORF, PCR amplified from indicated genomic DNA shows the BspE1 restriction site is lost in *SUI3* amplified from *sif285-1* cells.

Supplementary Fig. S3 eIF2 purification and interaction with eIF5.

A. Instant blue stained SDS-PAGE gel showing purified eIF2 and eIF2βE189K. **B.** Interaction between eIF2 and GST-eIF5 via GST-pulldown interaction assay. Bound eIF2 was probed using specific antibodies for eIF2α and eIF2γ and quantitative IR Western blot detection was performed using IRDye 800CW goat anti-rabbit IgG with an Odyssey Fc imaging system (Li-Cor). Proteins also detected with Instant Blue staining (Expedeon Ltd).

Supplementary Figure S1

A

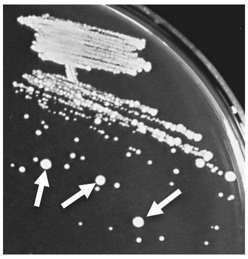

gcd6-F250L

B

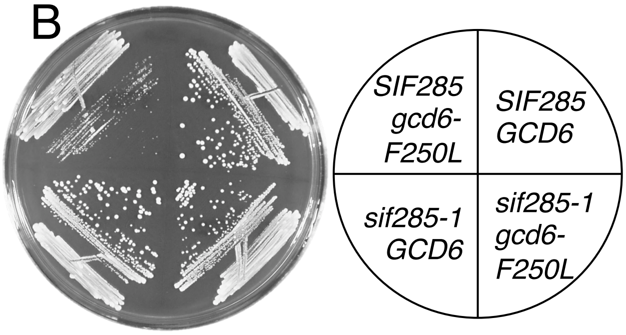

C

YPD, Day 2 WT sif285-1

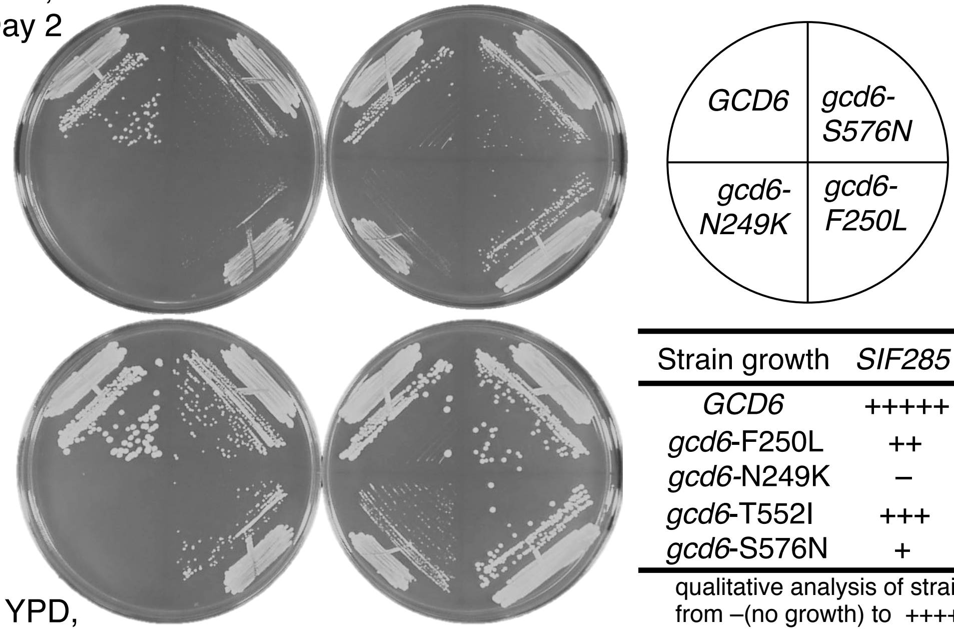

YPD, Day 3 WT sif285-1

Strain growth	*SIF285*	*sif285-1*
GCD6	+++++	+++++
gcd6-F250L	++	++++
gcd6-N249K	–	+
gcd6-T552I	+++	++++
gcd6-S576N	+	+++

qualitative analysis of strain growth rates: from –(no growth) to +++++ (WT growth)

D

YPD, Day 3

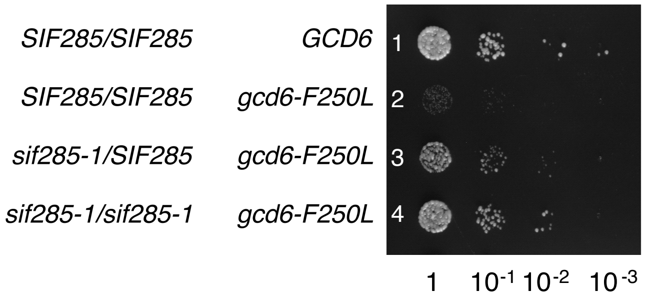

E *MAT***a**/α *SIF285/sif285-1 gcd6Δ/Δ* p[*gcd6-F250L*]

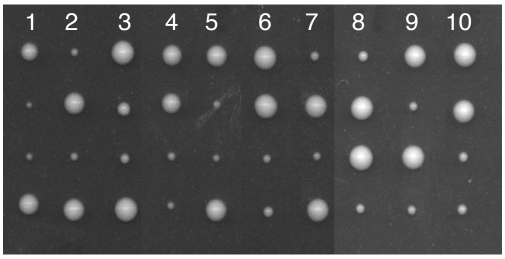

Supplementary Fig. S2

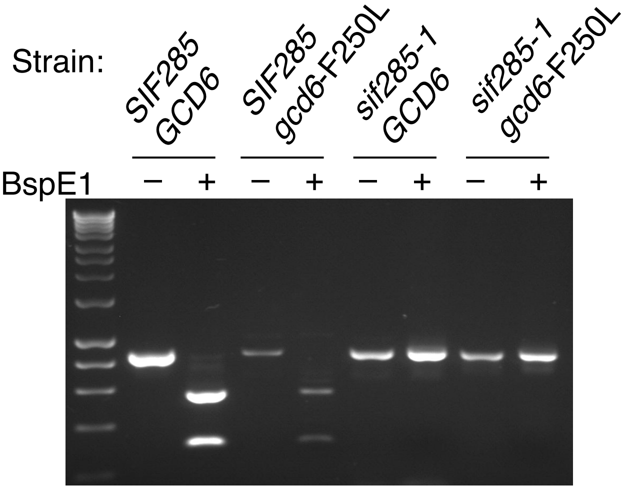

Supplementary Figure S3

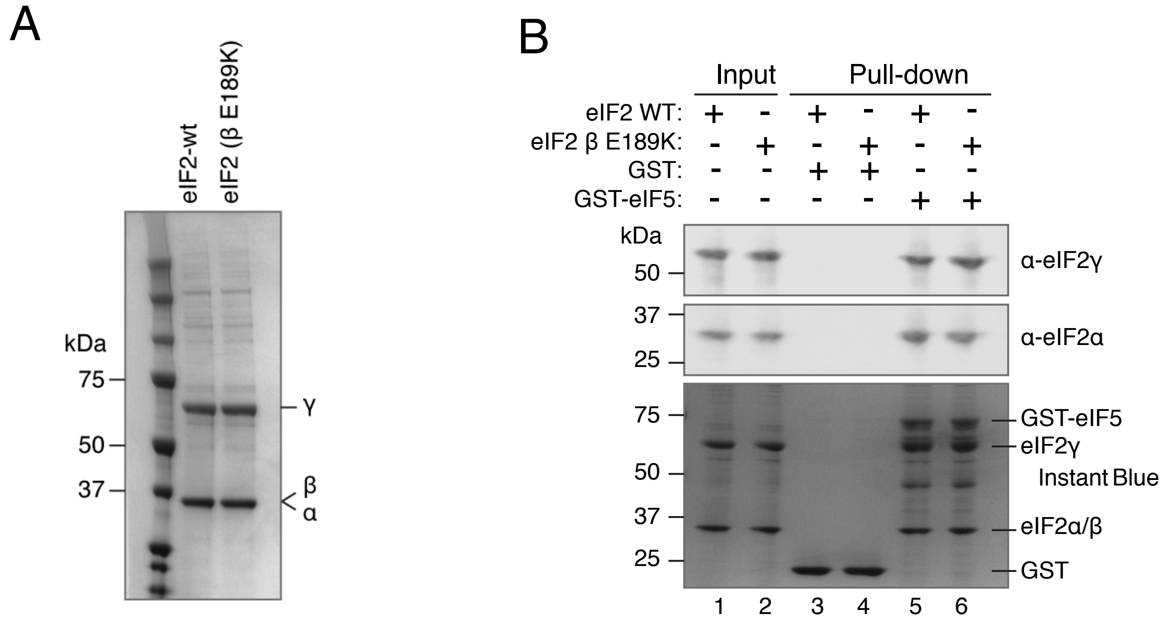

Supplement: SUPPLEMENTARY DATA [file supp_gkw657_nar-00474-m-2016-File008.pdf]
